# Precise and Rapid Validation of Candidate Gene by Allele Specific Knockout With CRISPR/Cas9 in Wild Mice

**DOI:** 10.3389/fgene.2019.00124

**Published:** 2019-02-19

**Authors:** Tianzhu Chao, Zhuangzhuang Liu, Yu Zhang, Lichen Zhang, Rong Huang, Le He, Yanrong Gu, Zhijun Chen, Qianqian Zheng, Lijin Shi, Wenping Zheng, Xinhui Qi, Eryan Kong, Zhongjian Zhang, Toby Lawrence, Yinming Liang, Liaoxun Lu

**Affiliations:** ^1^Institute of Psychiatry and Neuroscience, Xinxiang Medical University, Xinxiang, China; ^2^Henan Key Laboratory of Immunology and Targeted Therapy, School of Laboratory Medicine, Xinxiang Medical University, Xinxiang, China; ^3^Laboratory of Genetic Regulators in the Immune System, Henan Collaborative Innovation Center of Molecular Diagnosis and Laboratory Medicine, School of Laboratory Medicine, Xinxiang Medical University, Xinxiang, China; ^4^Centre for Inflammation Biology and Cancer Immunology, King’s College London, London, United Kingdom

**Keywords:** CD44, allele specific knockout, CRISPR/Cas9, functional genomics, wild mice

## Abstract

It is a tempting goal to identify causative genes underlying phenotypic differences among inbred strains of mice, which is a huge reservoir of genetic resources to understand mammalian pathophysiology. In particular, the wild-derived mouse strains harbor enormous genetic variations that have been acquired during evolutionary divergence over 100s of 1000s of years. However, validating the genetic variation in non-classical strains was extremely difficult, until the advent of CRISPR/Cas9 genome editing tools. In this study, we first describe a T cell phenotype in both wild-derived PWD/PhJ parental mice and F1 hybrids, from a cross to C57BL/6 (B6) mice, and we isolate a genetic locus on Chr2, using linkage mapping and chromosome substitution mice. Importantly, we validate the identification of the functional gene controlling this T cell phenotype, *Cd44*, by allele specific knockout of the PWD copy, leaving the B6 copy completely intact. Our experiments using F1 mice with a dominant phenotype, allowed rapid validation of candidate genes by designing sgRNA PAM sequences that only target the DNA of the PWD genome. We obtained 10 animals derived from B6 eggs fertilized with PWD sperm cells which were subjected to microinjection of CRISPR/Cas9 gene targeting machinery. In the newborns of F1 hybrids, 80% (*n* = 10) had allele specific knockout of the candidate gene *Cd44* of PWD origin, and no mice showed mistargeting of the B6 copy. In the resultant allele-specific knockout F1 mice, we observe full recovery of T cell phenotype. Therefore, our study provided a precise and rapid approach to functionally validate genes that could facilitate gene discovery in classic mouse genetics. More importantly, as we succeeded in genetic manipulation of mice, allele specific knockout could provide the possibility to inactivate disease alleles while keeping the normal allele of the gene intact in human cells.

## Introduction

Wild mice refer to both inbred lines and individual animals from natural house mouse populations, both of which harbor enormous genetic variations. Such models are particularly useful for study of genetic and environmental factors contributing to host immune response to pathogens (Rosshart et al., 2017). C57BL/6 and PWD/Ph strains are wild-derived mouse strains representing two major subspecies of house mouse, namely *M. m. domesticus* and *M. m. musculus* (Guenet and Bonhomme, 2003). The *M. m. domesticus* derived B6 mice and *M. m. musculus* derived PWK mice have highly diverged genomes with over 17 million single nucleotide polymorphisms (SNPs) which far outnumbers the genetic variations between classical laboratory strains (Keane et al., 2011). Over 90% of the genomic composition of classical laboratory mouse strains are mainly derived from subspecies *M. m. domesticus*, and remnants of the *M. m. musculus* genome are extremely rare (Yang et al., 2011). Therefore, from a genetics perspective, it is interesting to analyze phenotype of the *M. m. musculus* derived strain and harness the functional genetic variations which could not be found in classical mice (Gregorova and Forejt, 2000). We compared phenotype of T lymphocytes between B6 and PWD strains in an attempt to search for genetic factors contributing to T cell biology, which is essential to understand host defense against infection and cancer (Malissen and Bongrand, 2015). We found that a typical subset of naïve CD4 T cells expressing high levels of CD62L and low levels of CD44, was absent in the PWD strain. Both CD4 and CD8 T cells express higher levels of CD44 on cell surface in PWD mice. To map the genetic factor(s) responsible for this phenotype, we crossed B6 and PWD mice to generate F1 hybrids, interestingly the F1 mice had an identical phenotype to PWD mice, suggesting a dominant effect of these gene(s). We then backcrossed F1 mice to B6 to segregate the causative genetic alleles. Indeed, in the backcrossed population, we found 50% mice (275 out of 559) displaying the PWD T cell phenotype.

By means of genome wide scanning with genetic markers, we identified a single locus on Chr2 of PWD mice. Since the PWD strain was involved in a very particular genetic resource, named chromosome substitution strains, which are available for rapid validation of genetic mapping, we used the C57BL/6J-Chr2^PWD/Ph^/ForeJ strain which carries the entire Chr2 from PWD on the pure B6 background (Gregorova et al., 2008). We found that such B6.PWD-Chr2 mice have an identical T cell phenotype to PWD mice. In further fine mapping experiments, we found *Cd44 per se* was among the candidate genes in the mapped locus which was responsible for the T cell phenotype. CD44 is a cell surface marker for memory T cells and regulates memory cell survival (Baaten et al., 2010). In further experiments, we sought to inactivate the PWD derived Cd44 locus in F1 hybrids via CRISPR/Cas9 genome editing, as previous studies showed that PAM sequences were necessary to cleave target DNA and allele specific modification of DNA sequence could be performed in mice (Hsu et al., 2013; Wu et al., 2013). To perform functional gene validation involving wild mice in our study, we employed allele specific genome editing which was also reported in human iPSCs and rats, we first analyzed the sequences between PWD mice and B6 mice (Yoshimi et al., 2014; Smith et al., 2015). In the coding sequence of *Cd44*, two SNPs constitute CRISPR/Cas9 PAM sequences only for PWD mice which enables allele specific knockout of *Cd44*. Therefore, we could validate the functional relevance of this gene in F1 hybrids by specific knockout of the PWD allele. We designed two sgRNAs which were co-injected into B6 eggs fertilized by PWD sperms along with the CRISPR/Cas9 machinery. The results showed that among the 10 newborns, 80% of the mice carried ORF loss mutations of *Cd44* from PWD origin, with no mistargeting of the B6 *Cd44* allele. The CRISPR/Cas9 engineered F1 mutant mice had a phenotype identical to B6 mice. Therefore, we validated the causative gene for this phenotype, and our study provided a strategy for functional gene identification via allele-specific knockout, which could be useful for forward genetic studies in mice.

## Materials and Methods

### Animals

PWD/PhJ and Chromosome substitution mice C57BL/6J-Chr 2^PWD/Ph^/ForeJ strain (B6.PWD-Chr2, Stock No: 005995) were purchased from the Jackson Laboratory ^1^ via distribution by Shanghai MAOSHENGYAN Biologic Science & Technology Co., Ltd., ICR outbred foster mice and C57BL/6 mice were purchased from Beijing Vital River Laboratory Animal Technology Co., Ltd., and all animal procedures were performed according to guidelines approved by the committee on animal care at Xinxiang Medical University.

### Generation of CD44 Allele Specific Knockout F1 Mice

Single nucleotide polymorphisms or SNPs information of *Cd44* gene between PWK and C57BL/6 mice were analyzed from Mouse Genomes Project^2^. The SNPs were validated in PWD mice by Sanger sequencing before design of allele specific knockout sgRNAs. The sgRNA (Shao et al., 2014) and Cas9 mRNA (Liang et al., 2015) were produced by *in vitro* transcription (IVT) as descripted previously. 4-week old female C57BL/6N mice were intra-peritoneally injected Pregnant Mare Serum Gonadotropin (PMSG) at 5 p.m. followed by Human Chorionic Gonadotropin (hCG, 10 units/mouse) 48 h later and then mated with 12-week old male PWD mice immediately. Fertilized embryos were collected in the next morning and Cas9 mRNA (50 ng/μL) and sgRNA (50 ng/μL) were microinjected into the cytoplasm of fertilized embryos by using a standard microinjection system (Eppendorf TransferMan^®^ 4r, Eppendorf, Germany). Survived eggs were cultured at 37°C in 5% CO2 over-night and in the next day were transferred into the oviductal ampullas of the surrogate ICR mice.

Genomic DNA of F1 newborn tails were subjected to PCR analysis by using Phusion High-Fidelity DNA Polymerase (Thermo Fisher Scientific) with 5′-FAM-labeled primers to amplify two loci targeted by sgRNAs (Primer pair 1: GCTTTCTGGGGTGCTCTTCT; AGAGTATGTGGGTGAAGGGG. Primer pair 2: TGGATGTGAGATTGGGTCGAAG; GGCAGCATGTGTCGAGAATTAC). The PCR products were run on an ABI 3730 DNA analyzer and data analyzed by GeneMapper software V3.1. The positions of the peaks indicate the lengths of PCR products (Velasco et al., 2007). For sequencing, PCR products were further cloned into T-vector (Tiangen, China). In general, 10 colonies were picked from each agar plate and were proceeded to Sanger sequencing.

### Immunophenotyping by Flow Cytometry

The splenocytes and thymocytes of mice were stained with monoclonal antibody mixture and analyzed by flow cytometry. For activated T cell analysis, *in vitro* stimulation with anti-CD3 (3 μg/mL, 145-2C11, BD Biosciences) and anti-CD28 (1 μg/mL, 37.51, BD Biosciences) was performed in total splenocytes. The antibody labeling experiments were done as described in our previous studies for mouse immunophenotyping (Liang et al., 2013). In brief for splenocytes, 1 million cells were stained in 50 μL with antibody mixes CD3 (Alexa700), CD4 (eflour450), CD8 (PE), CD25 (PE-Cy5.5), CD44 (Brilliant Violet 605), CD45 (APC-eflour780), CD62L (APC), and TCRβ (FITC). Live cells were gated by Sytox blue staining and acquired on the FACS Canto flow cytometer (BD, United States). For immunophenotyping of the thymocytes, 2 million cells were stained in 50 μL with antibody mixture CD4 (PE), CD8 (Brilliant Violet 421), CD25 (APC), CD44 (Brilliant Violet 605) and acquired on the FACS Canto flow cytometer (BD, United States). The FACS data was analyzed using Flowjo software version 10.0.

### QTL Mapping

The inbred strain C57BL/6 female mice and PWD/PhJ male mice were crossed to obtain F1 hybrids. The resultant F1 mice were crossed to C57BL/6 mice to establish N2 backcross population for linkage mapping. Hundred and twenty short random repeat (STR) markers were used to construct a genetic map using the function est.map from the R/qtl package. The phenotypic data and genetic map were analyzed with R/qtl using a standard interval mapping method (Broman et al., 2003). Initial interval mapping was performed with the R/qtl function scannone using EM algorithm (Lander and Botstein, 1989), and a significant threshold at *p* = 0.05 for the selected T cell trait was determined with 1000 permutation. Next, refining the localization of QTL with logarithm of odds (LOD) score above threshold. To fine-map the major QTL implicated in mouse T cell phenotype, CD44 expression in CD4 T cells, we selected markers flanking the candidate gene on Chr2, 3 STR markers D2Mit75, D2Mit97 and D2Mit100 were used to screen the N2 individuals for recombinants with 491 mice. Then 37 recombinant mice were genotyped with 6 additional STR markers to narrow the mapped interval. The primers of the markers used for genotyping are listed in Supplementary Table S2.

### Statistical Analysis

GraphPad Prism software (version 7.0) was used for data analysis and statistical significance was assessed by unpaired, two-tailed Student’s *t*-test. Data were presented as Mean ± SEM. ^∗^*p* < 0.05, ^∗∗^*p* < 0.01, ^∗∗∗^*p* < 0.001, ^∗∗∗∗^*p* < 0.0001.

## Results

### Wild Derived PWD/PhJ Mice Display a Distinct T Cell Phenotype From C57BL/6 Mice

**FIGURE 1:**
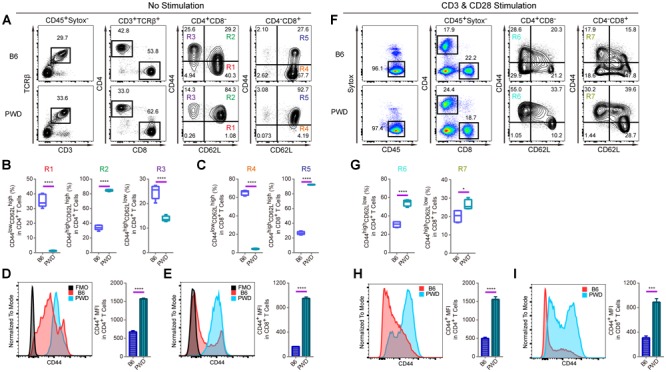
T cell phenotyping of B6 and PWD mice with splenocytes. For steady state analysis of splenocytes *ex vivo*, surface staining with antibodies labeling CD3 and TCRβ was used to define T cells in which CD4 and CD8 T cell subsets were further analyzed. For activated T cell analysis, *in vitro* stimulation with anti-CD3 and anti-CD28 was performed in total splenocytes. CD4 and CD8 T cells were analyzed for naïve and memory T cell frequencies in percentage and mean fluorescence intensity or MFI. **(A)** The gating method for T cells in the spleen of B6 and PWD mice. **(B)** Frequency of CD44^low^CD62L^high^ cells (R1), CD44^high^CD62L^high^ cells (R2) and CD44^high^CD62L^low^ cells (R3) in CD4 T cells (CD4^+^CD8^-^ cells) of B6 and PWD mice. **(C)** Frequency of CD44^low^CD62L^high^ cells (R4) and CD44^high^CD62L^high^ cells (R5) in CD8 T cells (CD4^-^CD8^+^ cells) of B6 and PWD mice. **(D)** MFI of CD44 in CD4 T cells of B6 and PWD mice, FMO control was used as negative control by staining all the surface labeling antibodies except CD44. **(E)** MFI of CD44 in CD8 T cells of B6 and PWD mice, FMO control was used as negative control by staining all the surface labeling antibodies except CD44. **(F)** The gating method for immune cells in the spleen of B6 and PWD mice after anti-CD3 (3 μg/mL, coated) and anti-CD28 (1 μg/mL, soluble) stimulation. **(G)** Frequency of CD44^high^CD62L^low^ cells in CD4 T cells (R6) and CD8 T cells (R7) of B6 and PWD mice after anti-CD3 (3 μg/mL, coated) and anti-CD28 (1 μg/mL, soluble) stimulation. **(H)** MFI of CD44 in CD4 T cells of B6 and PWD mice after anti-CD3 (3 μg/mL, coated) and anti-CD28 (1 μg/mL, soluble) stimulation. **(I)** MFI of CD44 positive cells in CD8 T cells of B6 and PWD mice after anti-CD3 (3 μg/mL, coated) and anti-CD28 (1 μg/mL, soluble) stimulation. Representative FACS data were from two independent experiments involving at least six animals for each group of mice. Data were analyzed by two-tailed Student’s *t*-test. Data were presented as Mean ± SEM. ^∗^*p* < 0.05, ^∗∗∗^*p* < 0.001, ^∗∗∗∗^*p* < 0.0001.

T lymphocytes are pivotal components in the immune system of mammals to fight against pathogens and tumors and their development is under sophisticated molecular control which requires genetic dissection (Liang et al., 2013; Wang et al., 2017). In both CD4 and CD8 T cells, which are the two major subsets of T lymphocytes, CD44 expression on the cell surface is used as important marker to monitor T cell activation (Huet et al., 1989; DeGrendele et al., 1997). In a standard B6 mouse, assessment of CD44 expression on the cell surface helps define activated memory T cells. *In vitro*, T cell receptor (TCR) stimulation with anti-CD3 and anti-CD28, results in high CD44 expression (Flaherty and Reynolds, 2015). Interestingly, T cells from PWD mice displayed a distinct CD44 expression pattern in steady-state compared to B6 mice, and an increase of effector memory T cells upon TCR (CD3 and CD28) stimulation. As shown in Figure 1A,B, the CD44^high^CD62L^high^ compartment among CD4-positive T cells, termed central memory cells (R2), was strikingly increased in PWD mice compared to B6 counterparts. While CD44^low^CD62L^high^ naïve T cells (R1), conversely, were dramatically reduced. In addition, age matched PWD mice had significantly less CD44^high^CD62L^low^ effector memory CD4 T cells (R3). Among CD8 T cells of PWD mice, the CD44^high^CD62L^high^ compartment was also dramatically increased in frequency (R5), while CD44^low^CD62L^high^ naïve T cells (R4), were again significantly reduced (Figure 1C). Next, we compared the mean fluorescent intensity (MFI) of CD44 by surface staining of CD4 and CD8 T cells; the MFI of CD44 in PWD CD4 T cells was 2.3-fold higher than in B6 mice, and in CD8 T cells CD44 expression was increased 5.8-fold (Figure 1D,E). Since CD44 expression can be induced by TCR stimulation, we analyzed the CD44 levels of CD4 and CD8 T cells between PWD and B6 mice following TCR stimulation. Notably, CD4 and CD8 T cells from PWD and B6 mice both showed a dramatic increase of CD44 expression 24 h after TCR stimulation, which could facilitate T cell migration to sites of inflammation. PWD mice had more CD44^high^CD62L^low^ effector memory cells among both CD4 and CD8 T cells (R6, R7), following *in vitro* activation by TCR stimulation (Figure 1F,G). Unexpectedly, the MFI of CD44 on both CD4 and CD8 T cells was 3-fold higher in PWD mice following TCR stimulation (Figure 1H,I). These results showed that PWD mice had significantly higher expression of CD44 in steady-state, and more importantly, CD44 upregulation following TCR stimulation was more potent in T cells of PWD mice.

### The Splenic CD4 T Cell Phenotype of PWD Mice Is Dominant in F1 Hybrids

**FIGURE 2:**
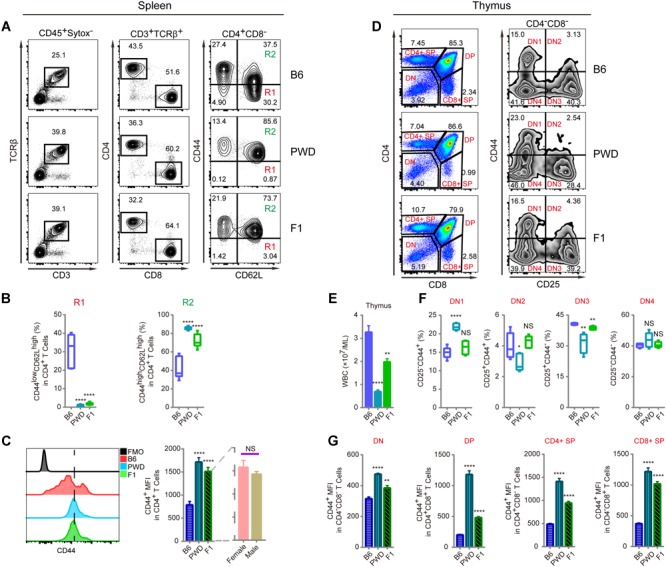
T cell phenotyping of B6, PWD and F1 mice for spleen and thymus. For peripheral T cell analysis, splenocytes were analyzed *ex vivo* for CD44 and CD62L expression in CD4 T cells. For thymocytes analysis, CD4 and CD8 T single positive T cells and their precursors namely CD4 and CD8 double positive and double negative cells were analyzed. Inside the double negative compartment, further analysis with CD25 and CD44 were used to define each precursor population. **(A)** The gating method for T cells in the spleen of B6, PWD and F1 mice. **(B)** Frequency of CD44^low^CD62L^high^ cells (R1) and CD44^high^CD62L^high^ cells (R2) in CD4^+^CD8^-^ cells of B6, PWD and F1 splenocytes. **(C)** Overlay of CD44 MFI in CD4 T cells of B6, PWD and F1 splenocytes, FMO control was used as negative control by staining all the surface labeling antibodies except CD44. **(D)** The gating method for immune cells in the thymus of B6, PWD and F1 mice. **(E)** Absolute thymocyte count of B6, PWD and F1 mice. **(F)** Frequency of DN1-DN4 cells in DN cells of B6, PWD and F1 thymocytes. **(G)** MFI of CD44 positive cells in DN, DP, CD4^+^ SP and CD8^+^ SP cells of B6, PWD and F1 thymocytes. Representative FACS data were from two independent experiments involving at least seven animals for each group of mice. Data were analyzed by two-tailed Student’s *t*-test. Data were presented as Mean ± SEM. ^∗∗^*p* < 0.01, ^∗∗∗∗^*p* < 0.0001.

Since CD44 is implicated in T cell mobilization and differentiation into immunotolerant T cells, we analyzed the inheritance pattern of the T cell phenotype observed in PWD mice, prior to genetic mapping of the chromosomal region containing the causative gene(s) (DeGrendele et al., 1997; Wu et al., 2014). In F1 hybrids derived from B6 and PWD crosses, the CD4 T cells had significantly higher CD44 expression in *ex vivo* assays, closely resembling the phenotype of parental PWD mice with respect to R1 and R2 compartments, shown in Figure 2A. Furthermore, both F1 and PWD parental strains had dramatically different distributions of naïve and central memory T cells (Figure 2B). CD44 expression by CD4 T cells, was significantly higher in the PWD parental strain and F1 mice than that of B6 mice, and this increase in CD44 was not distinguishable between male and female F1 mice (Figure 2C). Since the increased CD44 expression on CD4 T cells was observed in both F1 and parental PWD mice, we regard this phenotype as “dominant,” even though the phenotype in CD8 T cells was less stringent (data not shown). We further analyzed the CD44 expression in F1 hybrids in thymocytes which give rise to T cells. CD4^-^CD8^-^double negative (DN) thymocytes, which are the progenitors of T cells, cells were further divided into DN1, DN2, DN3, and DN4 populations (Figure 2D). As shown in Figure 2E,F, F1 mice either displayed an intermediate phenotype, between the two parental strains of B6 and PWD, or no significant difference from B6 mice, for multiple parameters including the thymocyte number and CD44 expression. In more complete comparisons between F1 and parental mice for CD44 expression in various populations in thymus, we found that F1 phenotype was generally intermediate (Figure 2G). These results indicated that the dominant PWD phenotype in CD4 T cells of increased CD44 expression, was restricted to T cells in the periphery. Therefore, we used CD4 T cell phenotype to perform further genetic mapping experiments to facilitate identification of causative gene.

### A Single Genetic Locus on Chr2 Controls the PWD CD4 T Cell Phenotype

**FIGURE 3:**
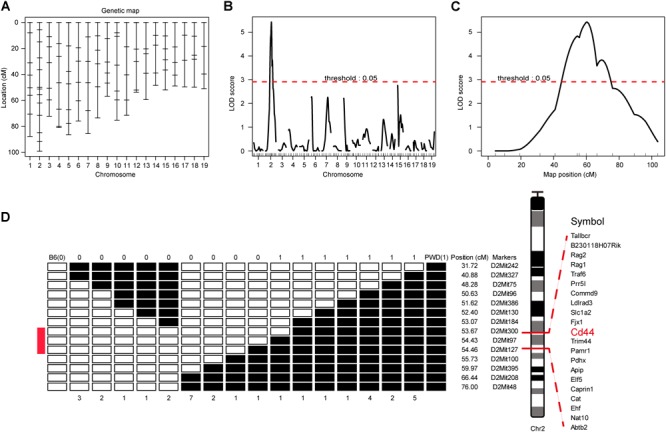
Genome-wide scanning to map QTL influencing the CD4 T phenotype. The dominant phenotype of CD4 T cells was used to categorize mice with PWD phenotype or B6 phenotype by absence or presence of the “naïve” T cells which were CD44^low^CD62L^high^ (refer to Figure 2A, R1). **(A)** The genetic maker distribution throughout the mouse genome to analyze N2 population with 120 STRs. **(B)** LOD score curves of the initial mapping with 68 animals (37 phenotypic mice, and 31 non-phenotypic mice), and chromosome 1–19 were represented numerically on the *X*-axis. The relative width of the space allotted for each chromosome reflects the number of microsatellite markers typed for that chromosome. The *Y*-axis represents the LOD score. The horizontal dashed lines denote genome-wide empirical thresholds for significant (*p* = 0.05) linkage. **(C)** Interval mapping identified one major QTL in the position from 47.42 to 66.42 cM on mouse chromosome 2. **(D)** Fine-mapping of the major Chr2 QTL in an N2 population of 491 individuals. Open box denotes B6 genotype of the STR markers, and filled box represents PWD genotype. “0” was used to represents B6 phenotype and “1” for PWD phenotype. Inside the fine mapped interval between D2Mit300 and D2Mit127, candidate genes were listed.

To map the genetic factor(s) regulating the T cell phenotype we observed in PWD mice, we constructed a backcross pedigree for linkage mapping by crossing F1 hybrids to B6 mice (Mihola et al., 2009; Su et al., 2009). In the resultant backcross N2 animals, we observed obvious phenotype segregation in 1:1 ratio, suggesting a single gene or closely linked genes were responsible for the phenotype. Among the 559 N2 mice, 275 mice had high CD44 expression, another 284 animals had low CD44 expression, sex ratio was close to 1:1. Genome wide scanning with 120 microsatellite markers was performed initially with 68 N2 mice (Figure 3A). We found the highest LOD score was located at Chr2 D2Mit395 (Figure 3B,C). From the chromosome substitution (CS) mouse resource or consomic mice, the C57BL/6J-Chr 2^PWD/Ph^/ForeJ (B6.PWD-Chr2, Stock No: 005995) strain carries a complete Chr2 from PWD and the rest of the genome is of C57BL/6 origin. In these CS mice we confirmed the T cell phenotype, originally found in PWD mice. In the B6.PWD-Chr2 CS strain, CD44 expression on CD4 T cells was comparable to the PWD mice and significantly higher than the B6 mice (Supplementary Figures S1A,B). Therefore, we mapped the genetic locus for this phenotype to Chr2, and further validated the localization of the causative locus in CS mice. Following initial linkage mapping, fine mapping using an additional 491 N2 mice, further identified that a 1 cM chromosomal segment between D2Mit300 and D2Mit127 was responsible for this phenotype (Figure 3D and Supplementary Table S1). Within this chromosomal segment, there were 21 protein coding genes. Referring to Immunological Genome Project database (Heng et al., 2008; Benoist et al., 2012), 8 genes were expressed in T cells which included Cd44, Cat, Caprin, Trim44, Ldlrad3, Traf6, Rag1, and Rag2 (Supplementary Table S1). We suspected *Cd44* as the candidate gene, since the T cell phenotype was defined by changes in CD44 expression, and more notably among the candidate genes only Cd44 mRNA expression was higher in PWD CD4 T cells than the B6 controls (Immgen database, and data not shown).

### Allele Specific Knockout of PWD Cd44 in F1 Mice by CRISPR/Cas9

**FIGURE 4:**
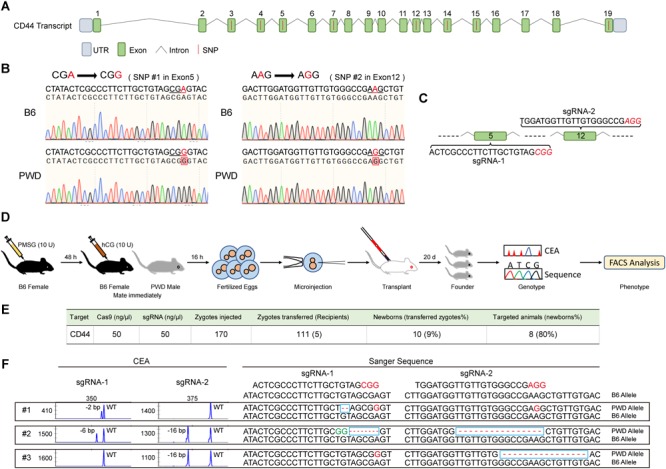
Generation and genotyping of PWD CD44 allele specific knockout in F1 mice. **(A)** The distribution of SNPs in *Cd44* exons of the whole genome sequenced PWK strain which is a close relative of PWD strain. **(B)** Two PAM sequences (5′-NGG-3′) that only existed in the PWD mice were selected and confirmed by Sanger sequencing. **(C)** SgRNA sequences in *Cd44* exon5 and exon12 which were selected for allele specific knockout. **(D)** Workflow of producing PWD CD44 allele specific knockout in F1 mice. **(E)** Efficiency of CRISPR/Cas9 system in specific inactivation of CD44 from PWD origin. **(F)** The genotyping of F1-CD44*^ASK^* mice by using capillary electrophoresis analysis (left) and Sanger sequencing (right).

We suspected that *Cd44 per* se was responsible for the dominant T cell phenotype in PWD and F1 mice, based on fine mapping which resulted in 21 candidate genes and 2 differentially expressed genes between the parental strains (Supplementary Table S1). To confirm the dominant effect of the *Cd44* allele originating from PWD mice on the T cell phenotype we observed in F1 mice, we designed allele specific knockout (ASK) to inactivate only the PWD copy of *Cd44* gene. CRISPR/Cas9 genome editing requires guide RNA and PAM sequence to target specific DNA elements. In the absence of PAM, genome editing efficiency was not detectable. To validate that PWD *Cd44* itself determines the T cell phenotype we observed in B6/PWD F1 hybrids, we sought to inactivate only the PWD copy of the gene, since the phenotype was dominant. The exon and intron structure of murine *Cd44* and SNPs between B6 and PWK, which is closely related to PWD, are depicted in Figure 4A. The whole genome sequence and SNPs for PWK, but not PWD, mice are available from the Mouse Genomes Project ^3^. The CD44 protein coding sequences of B6 and PWK were aligned and 8 out of 19 exons had SNPs (Figure 4A). Among these SNPs we selected those that formed PAM (5′-NGG-3′) sequences which were only existing in the PWK mice, and such SNPs were later confirmed in PWD mice by Sanger sequencing (Figure 4B). The PWD copy of *Cd44* were sequenced for cDNA, and we found 2 amino acid absence in comparison to B6 protein sequence (Supplementary Data Sheet S1). The guide RNA was prepared by IVT, and potential off-targets were analyzed using the CRISPOR software, as described previously (Luo et al., 2018). Before microinjection of the CRISPR/Cas9 machinery designed to specifically target *Cd44* of PWD mice, fertilized eggs were prepared by superovulation of B6 female mice aged 4–6 weeks and fertilization with sperm cells from PWD male mice aged 10–12 weeks. Two sets of allele specific guide RNAs and Cas9 mRNA were co-injected (Figure 4C). The engineered eggs were developed in ICR outbred foster mice and genotyped using mouse tail tip DNA from newborns by fluorescent PCR and capillary gel electrophoresis (Figure 4D). As shown in Figure 4E, in total 170 fertilized eggs were subject to microinjection and 111 live eggs were transplanted, resulting in 8 mice with Indels. In theory, the Indels should only occur for the PWD allele of *Cd44*, which possesses PAM sequences that are absent in B6 mice. To validate the consequence of genome editing by allele specific targeting, we sequenced all the DNA samples from mice which carried Indel mutations by TA cloning of the PCR products and Sanger sequencing (Luo et al., 2018). Indeed, the Indel mutations were stringently restricted to the PWD allele, and all the B6 alleles tested were completely intact (Figure 4F and Supplementary Table S3). By selecting PAM sequences specific to the allele of PWD, we obtained animals carrying only the B6 copy of *Cd44* gene in F1 hybrids which were used for further phenotyping.

### Allele Specific Knockout of PWD Cd44 Rescues the T Cell Phenotype in Hybrid F1 Mice

**FIGURE 5:**
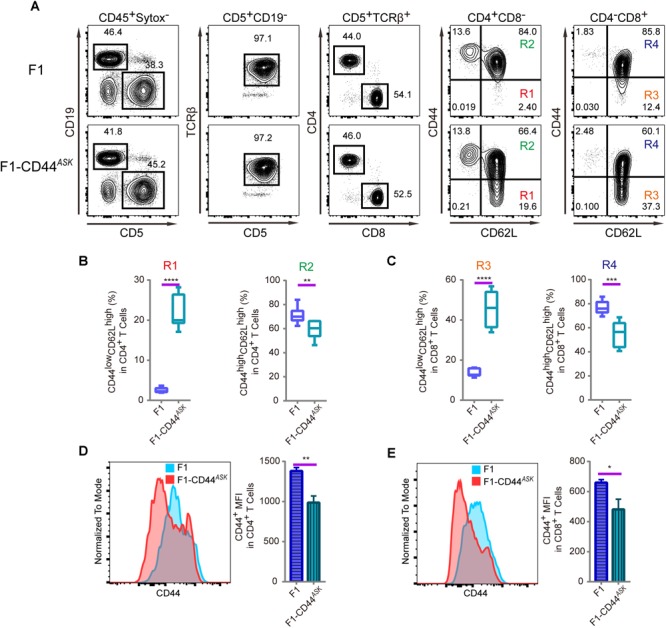
Immunophenotyping of T cells in splenocytes of F1 and F1-CD44*^ASK^* mice. In the F1 mice, consequence of the allele specific knockout of CD44 was analyzed by assessing the alteration of phenotype which was observed in PWD parental strain. **(A)** The gating method for T cells in the spleen of F1 and F1-CD44*^ASK^* mice. **(B)** Frequency of CD44^low^CD62L^high^ cells (R1) and CD44^high^CD62L^high^ cells (R2) in CD4^+^CD8^-^ cells of F1 and F1-CD44*^ASK^* mice. **(C)** Frequency of CD44^low^CD62L^high^ cells (R3) and CD44^high^CD62L^high^ cells (R4) in CD4^-^CD8^+^ cells of F1 and F1-CD44*^ASK^* mice. **(D)** MFI of CD44 positive cells in CD4^+^ cells of F1 and F1-CD44*^ASK^* mice. **(E)** MFI of CD44 positive cells in CD8^+^ cells of F1 and F1-CD44*^ASK^* mice. Representative FACS data were from two independent experiments involving at least six mice for each genotype. Data were analyzed by two-tailed Student’s *t*-test. Data were presented as Mean ± SEM. ^∗^*p* < 0.05, ^∗∗^*p* < 0.01, ^∗∗∗^*p* < 0.001, ^∗∗∗∗^*p* < 0.0001.

The T cell phenotype we observed in PWD and F1 mice was comparable, furthermore, the phenotype was consistent in N2 backcross mice and distributed in mendelian ratio. We mapped the causative PWD gene to Chr2 and further validated the phenotype using chromosome substitution mice, which have a clean genomic background identical to B6 animals, except for the donor chromosome. CRISPR/Cas9 genome editing allowed us to obtain F1 animals that were specifically deficient in the *Cd44* allele of PWD origin. In such allele specific knockout (ASK) mice, we tested the T cell phenotype and found that the phenotype in ASK mice differed significantly from that of PWD and F1 mice, and resembled the B6 phenotype. As shown in Figure 5A–C, comparison of the T cell phenotype between F1 mice and *Cd44^ASK^* F1 mice for CD4 and CD8 subsets revealed that inactivation of PWD CD44 resulted in significant alteration, notably increased frequency of CD44^low^CD62L^high^ naïve CD4 (R1) and CD8 T cells (R3) and conversely, decrease of CD44^high^CD62L^high^ central memory CD4 (R2) and CD8 T cell (R4) frequencies. *Cd44^ASK^* F1 mice, that were deficient in only the PWD copy of *Cd44*, had significantly decreased CD44 MFI in both CD4 and CD8 T cells (Figure 5D,E). We performed further experiments to compare *Cd44^ASK^* F1 mice and B6 mice, interestingly both CD4 and CD8 T cells had decreased CD44 expression in allele specific knockout F1 mice, to a level comparable to B6 T cells (Supplementary Figures S2A–C). Therefore, allele specific knockout of PWD CD44 in F1 mice was sufficient to restore the T cell phenotype to that of parental B6 mice. This allele specific knockout strategy demonstrated that a candidate gene from a specific parental origin can be precisely targeted in hybrid mice.

## Discussion

Mouse genetics contributes enormously to understanding of human pathophysiology (Demant, 2003; Nguyen and Xu, 2008). Functional mirroring of human immune cells in mice has led to discovery of novel cellular and genetic components of the immune system (Liang et al., 2013; Wang et al., 2016). Gene discovery via forward genetic mapping in mice has been highly successful in the last three decades, as genome wide scanning revealed thousands of genetic loci implicated in various diseases and immunological traits (Peters et al., 2007; Siggs, 2014). However, identification of the causative gene in the mapped loci, remains extremely difficult, due to extensive linkage disequilibrium and low efficiency in validation of candidate genes (Peters et al., 2007). The development of chromosome substitution mice provides a straight forward and rapid method for validation of mapped loci, maintaining a homogeneous genomic background identical to the reference C57BL/6 mice, however, cloning the causative gene is still challenging. In this study, we first discovered a T cell phenotype which was dramatically different between B6 and PWD mice, and further mapped locus on Chr2 responsible for the phenotypic difference. To validate the causative role of the mapped locus, we analyzed the chromosome substitution line B6.PWD-Chr2 and found the same phenotype as observed in PWD parental mice. Such results excluded genetic factors outside Chr2 that could co-contribute to the T cell phenotype we observed in PWD mice.

Fine mapping can be achieved with the N2 progeny by increasing the number of samples, to analyze more recombinants, or increasing the generations of backcrossing to generate more recombination flanking the causative gene(s) (Jeffs et al., 2000). In the initial mapping of the dominant locus, we used 68 mice, and for fine mapping we analyzed 491 animals that gave rise to an interval confining the causative gene inside a 1 cM segment on Chr2 of PWD strain. Among the candidate genes, we highly suspected *Cd44* itself as causative gene, as the CD44 protein was more abundantly expressed in CD4 T cells of PWD mice (Heng et al., 2008). Our experiments reflect numerous other studies using forward genetics in mice to identify genetic variation in inbred lines based on phenotyping and to test for candidate genes via linkage mapping, however, the challenge to confirm the role of the PWD allele in regulating T cell phenotype lied in obtaining selective knockout of this allele and test its consequence on a background that should otherwise maintain the phenotype. Knockout of the gene on a B6 background does not provide direct evidence of the functional role, since B6 mice themselves do not have the PWD phenotype. We found that F1 hybrids keep the PWD phenotype, therefore we could test by specific CRISPR/Cas9 mediated targeting of PWD allele of candidate gene *Cd44*.

We have established a procedure for producing allele specific knockout of a candidate gene by designing sgRNA with PAM sequences that only exist on the PWD background. Even though both copies of the gene from B6 and PWD parents were exposed to CRISPR/Cas9 machinery, precise and allele specific targeting of only the PWD allele of *Cd44* was achieved in our study, and it is interesting to note that such experiments could be further improved by applying variants of Cas9 nuclease to obtain higher fidelity in genome editing and non-canonical PAM sequences could be applied to fit broader range of genomic contexts (Zhang et al., 2014; Kleinstiver et al., 2016) In the F1 animals which were mutated only in the PWD copy of *Cd44* had an altered phenotype compared to B6 parental mice. Therefore, by allele specific targeting of PWD CD44, we confirmed the functional role of CD44 itself in maintaining the CD44^high^CD62L^high^ population only found in PWD mice. The molecular mechanisms behind how PWD CD44 contributes to this T cell phenotype, still remain to be elucidated, which could provide further hints to uncover new roles for CD44 since this molecule has been already found crucial for regulatory T cell development (Wu et al., 2014). More importantly, our approach of allele specific knockout could provide a new strategy to inactivate disease alleles while keeping the normal allele of the gene intact.

## Data Availability Statement

All datasets for this study are included in the manuscript and the Supplementary Files.

## Author Contributions

YL designed the project and wrote the manuscript. LL supervised the project, analyzed the data, and prepared the figures. TC and ZL performed the experiments and analyzed the data. YZ established the N2 population and performed the initial QTL mapping. ZL, RH, LH, YG, ZC, QZ, LS, WZ, and XQ were involved in preparation of mRNA, mouse embryos and genotyping. EK and ZZ assisted in supervising the project. TL contributed to writing and revising the manuscript. All authors read and approved the final manuscript.

## Conflict of Interest Statement

The authors declare that the research was conducted in the absence of any commercial or financial relationships that could be construed as a potential conflict of interest.

## Footnotes

1 www.jax.org

2 https://www.sanger.ac.uk/science/data/mouse-genomes-project

3 www.sanger.ac.uk/sanger/Mouse_SnpViewer
