# Supplementary material for: Precise and Rapid Validation of Candidate Gene by Allele Specific Knockout With CRISPR/Cas9 in Wild Mice
Source: Front Genet. 2019 Feb 19;10:124. doi: 10.3389/fgene.2019.00124 (PMC6390232; doi:10.3389/fgene.2019.00124)
Supplement: Supplementary file 4 [file Presentation_1.PPTX]

## Slide 1
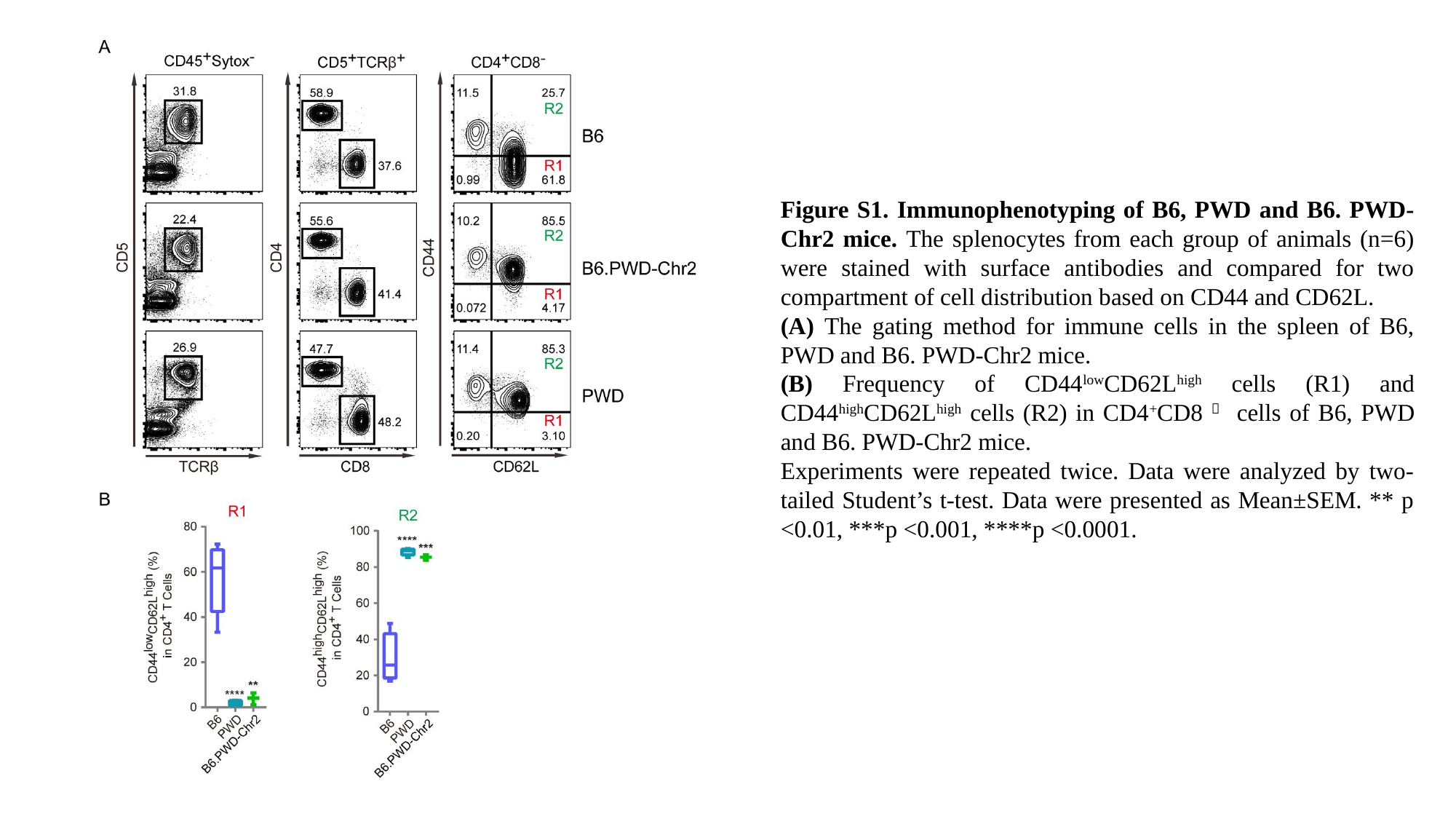

Figure S1. Immunophenotyping of B6, PWD and B6. PWD-Chr2 mice. The splenocytes from each group of animals (n=6) were stained with surface antibodies and compared for two compartment of cell distribution based on CD44 and CD62L.
(A) The gating method for immune cells in the spleen of B6, PWD and B6. PWD-Chr2 mice.
(B) Frequency of CD44lowCD62Lhigh cells (R1) and CD44highCD62Lhigh cells (R2) in CD4+CD8－ cells of B6, PWD and B6. PWD-Chr2 mice.
Experiments were repeated twice. Data were analyzed by two-tailed Student’s t-test. Data were presented as Mean±SEM. ** p <0.01, ***p <0.001, ****p <0.0001.

## Slide 2
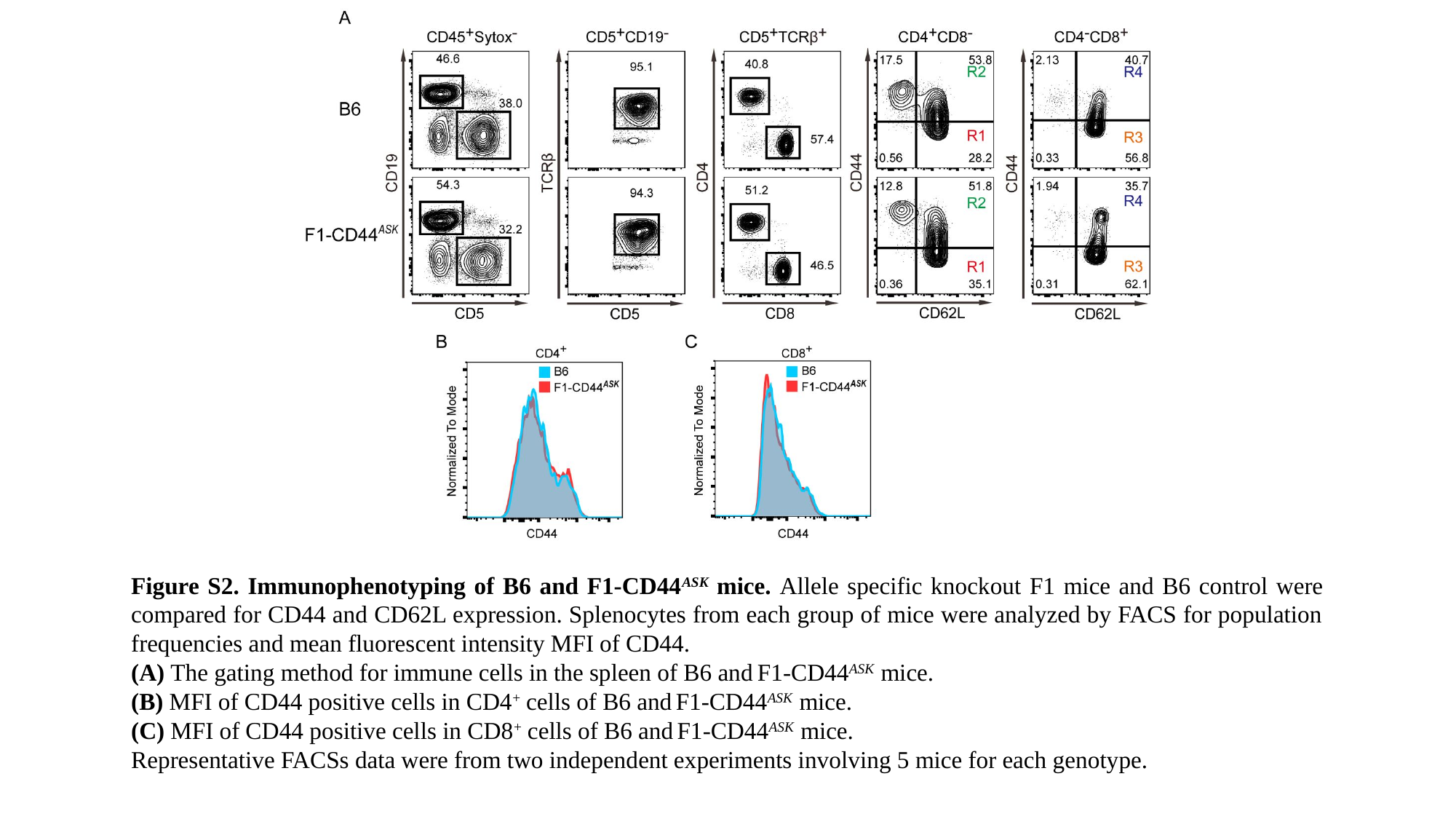

Figure S2. Immunophenotyping of B6 and F1-CD44ASK mice. Allele specific knockout F1 mice and B6 control were compared for CD44 and CD62L expression. Splenocytes from each group of mice were analyzed by FACS for population frequencies and mean fluorescent intensity MFI of CD44.
(A) The gating method for immune cells in the spleen of B6 and F1-CD44ASK mice.
(B) MFI of CD44 positive cells in CD4+ cells of B6 and F1-CD44ASK mice.
(C) MFI of CD44 positive cells in CD8+ cells of B6 and F1-CD44ASK mice.
Representative FACSs data were from two independent experiments involving 5 mice for each genotype.
