# Supplementary material for: Precise and Rapid Validation of Candidate Gene by Allele Specific Knockout With CRISPR/Cas9 in Wild Mice
Source: Front Genet. 2019 Feb 19;10:124. doi: 10.3389/fgene.2019.00124 (PMC6390232; doi:10.3389/fgene.2019.00124)
Supplement: Supplementary file 5 [file Data_Sheet_1.pdf]

Supplementary Datasheet 1. Alignment of amino acid sequences of CD44 between PWD and B6 mice

```
B6      MDKFWWHTAWGLCLLQLSLAHPHQQIDLNVTCRYAGVFHVEKNGRYSISRTEAADLCQAF   60
PWD     MDKFWWHTAWGLCLLQLSLA--HQQIDLNVTCRYAGVFHVEKNGRYSISRTEAADLCQAF   58
        ********************  **************************************

B6      NSTLPTMDQMKLALSKGFETCRYGFIEGNVVIPRIHPNAICAANHTGVYILVTSNTSHYD   120
PWD     NSTLPTMDQMKLALSKGFETCRYGFIEGNVVIPRIHPNAICAANHTGVYILVTSNTSHYD   118
        ************************************************************

B6      TYCFNASAPPEEDCTSVTDLPNSFDGPVTITIVNRDGTRYSKKGEYRTHQEDIDASNIID   180
PWD     TYCFNASAPPEEDCTSVTDLPNSFDGPVTITIVNRDGTRYSKKGEYRTHQEDIDASNIID   178
        ************************************************************

B6      DDVSSGSTIEKSTPESYILHTYLPTEQPTGDQDDSFFIRSTLATIASTVHSKSHAAAQKQ   240
PWD     DDVSSGSTIEKSTPESYILHTYLPTEQPTGDQDDSFFIRSTLATIASTVHSKSHAAAQKQ   238
        ************************************************************

B6      NNWIWSWFGNSQSTTQTQEPTTSATTALMTTPETPPKRQEAQNWFSWLFQPSESKSHLHT   300
PWD     NNWIWSWFGNSQSTTQTQEPTTSATTALMTTPETPPKRQEAQNWFSWLFQPSESKSHLHT   298
        ************************************************************

B6      TTKMPGTESNTNPTGWEPNEENEDETDKYPSFSGSGIDDDEDFISSTIASTPRVSARTED   360
PWD     TTKMPGTESNTNPTGWEPNEENEDETDKYPSFSGSGIDDDEDFISSTIASTPRVSARTED   358
        ************************************************************

B6      NQDWTQWKPNHSNPEVLLQTTTRMADIDRISTSAHGENWTPEPQPPFNNHEYQDEEETPH   420
PWD     NQDWTQWKPNHSNPEVLLQTTTRMADIDRISTSAHGENWTPEPQPPFNNHEYQDEEETPH   418
        ************************************************************

B6      ATSTTPNSTAEAAATQQETWFQNGWQGKNPPTPSEDSHVTEGTTASAHNNHPSQRITTQS   480
PWD     ATSTTPNSTAEAAATQQETWFQNGWQGKNPPTPSEDSHVTEGTTASAHNNHPSQRITTQS   478
        ************************************************************

B6      QEDVSWTDFFDPISHPMGQGHQTESKDTDSSHSTTLQPTAAPNTHLVEDLNRTGPLSVTT   540
PWD     QEDVSWTDFFDPISHPMGQGHQTESKDTDSSHSTTLQPTAAPNTHLVEDLNRTGPLSVTT   538
        ************************************************************

B6      PQSHSQNFSTLHGEPEEDENHPTTSILPSSTKSGAKDARRGGSLPTDTTTSVEGYTFQYP   600
PWD     PQSHSQNFSTLHGEPEEDENHPTTSILPSSTKSGAKDARRGGSLPTDTTTSVEGYTFQYP   598
        ************************************************************

B6      DTMENGTLFPVTPAKTEVFGETEVTLATDSNVNVDGSLPGDRDSSKDSRGSSRTVTHGSE   660
PWD     DTMENGTLFPVTPAKTEVFGETEVTLATDSNVNVDGSLPGDRDSSKDSRGSSRTVTHGSE   658
        ************************************************************

B6      LAGHSSANQDSGVTTTSGPMRRPQIPEWLIILASLLALALILAVCIAVNSRRRCGQKKKL   720
PWD     LAGHSSANQDSGVTTTSGPMRRPQIPEWLIILASLLALALILAVCIAVNSRRRCGQKKKL   718
        ************************************************************

B6      VINGGNGTVEDRKPSELNGEASKSQEMVHLVNKEPSETPDQCMTADETRNLQSVDMKIGV   780
PWD     VINGGNGTVEDRKPSELNGEASKSQEMVHLVNKEPSETPDQCMTADETRNLQSVDMKIGV   778
        ************************************************************

B6      *      780
PWD     *      778
        *
```
